# Engrailed-2 (EN2) protein in cervical mucus: a novel biomarker for endometrial carcinoma

**DOI:** 10.1007/s12094-024-03799-5

**Published:** 2024-11-28

**Authors:** Tong Wang, Ningyi Jia, Songkun Gao, Shengjie Liu, Ming Liu, Hong Zhang

**Affiliations:** 1 Department of Gynecological Oncology, Beijing Obstetrics and Gynecology Hospital, Capital Medical University, Beijing Maternal and Child Health Care Hospital, No.17 QiHeLou Street, Dongcheng District, Beijing, 100006 China; 2 Department of Urology, Beijing Hospital, No. 1 DaHua Road, Dongcheng District, Beijing, China; 3 Institute of Cardiovascular Sciences, Peking University Health Science Center, No. 38 Xueyuan Road, Handian District, Beijing, China; 4 National Center of Gerontology, No. 1 DaHua Road, Dongcheng District, Beijing, China

**Keywords:** Engrailed-2, Cervical mucus, Endometrial carcinoma, Biomarker, Diagnosis

## Abstract

**Objective:**

This study aims to demonstrate that the EN2 protein in cervical mucus may serve as a novel biomarker for screening endometrial cancer.

**Materials and Methods:**

This study included 133 patients who were treated at Beijing Obstetrics and Gynecology Hospital. According to the pathological results of hysteroscopy endometrial biopsy, the patients were divided into endometrial cancer group (*n* = 55), endometrial atypical hyperplasia group (*n* = 16), benign lesion group (*n* = 28), and control group (*n* = 34). All patients collected cervical mucus before hysteroscopy, and the level of EN2 protein in cervical mucus was detected by ELISA in the four groups of patients. Nine patients who underwent surgical treatment for endometrial cancer were included, and endometrial cancer lesions and adjacent tissues without endometrial cancer infiltration were taken from the endometrial cancer lesions and cervical tissues. Fifteen patients who underwent hysterectomy for benign lesions were included, and endometrial and cervical tissues were taken from the endometrial cancer lesions and adjacent tissues without endometrial cancer infiltration. PT-PCR, immunohistochemistry, and WB were used to verify the expression differences of EN2 protein in cancerous lesions and endometrial tissues without endometrial infiltration, cervical tissues, and endometrial tissues without endometrial lesions. In addition, HEC1B, KLE, and HeLa cell lines were used to characterize EN2 overexpression in endometrial cancer cells. Immunohistochemistry, RT-PCR, and confocal analysis were used to detect the expression of EN2 protein in different cell lines.

**Results:**

In different groups, the average concentration of EN2 protein in cervical mucus in the EC group was significantly higher than that in the benign group and the normal control group (573.9 ± 123.4 ng/mL vs 153.5 ± 106.2 ng/mL and 153.0 ± 107.5 ng/mL, *P* < 0.001). The concentration of EN2 protein in EIN3 case specimens was significantly higher than that in the normal control group (*P* < 0.001). ROC analysis showed that the optimal threshold for diagnosing endometrial cancer in cervical mucus was 321.1 ng/ml, with a sensitivity of 92.6% and specificity of 95.4% for distinguishing EINIII, EC, and non-cancerous cases. In clinical specimens, the expression level of EN2 protein in endometrial cancer tissues was significantly higher than that in endometrial tissues and cervical tissues without endometrial cancer infiltration. In addition, PT-PCR, immunohistochemistry, suggest that EN2 protein is overexpressed in endometrial cancer cell lines compared to cervical adenocarcinoma cells (*P* < 0.01).

**Conclusion:**

The concentration of EN2 protein in cervical mucus can effectively identify endometrial cancer and precancerous lesions. The increased expression of EN2 protein in endometrial cancer lesions leads to an increase in the concentration of EN2 protein in the cervical mucus of endometrial cancer patients.This is a small single-center study, and larger studies are needed to determine the role of EN2 protein in the diagnosis of endometrial cancer.

## Introduction

Endometrial carcinoma (EC) is one of the most common gynecological cancers. 382 069 new cases of corpus uteri cancer and 89 929 women died from the disease all over the world in 2018 [1]. 5-year survival for early-stage disease is 69–90%, while 5-year survival decreases to 17–58% for stages III-IV. Early detection of endometrial cancer and timely treatment can improve the survival rate of patients. Lack of efficient noninvasive screening methods for detecting early-stage endometrial cancer is always a problem worldwide. The most widely used tumor markers (such as Ca125 and HE4) are not suitable for endometrial cancer screening, especially at early stages [2–4].Because in the diagnosis of endometrial cancer the sensitivity of HE4, CA125, and combination of both were 66.0%, 33.0%, and 64.0% [10, 11]. The consensus strategy for screening endometrial cancer consists of transvaginal ultrasound (TVS) plus endometrial pipelle sampling in cases of abnormal endometrial thickness in some developed countries. However, such method is not an effective method for endometrial cancer screening. On one side, endometrial thickness measured by TVS is less specific for endometrial cancer and only used in postmenopausal women. It was referred that Only 7.3% of postmenopausal bleeding cases are endometrial cancer with an endometrial thickness threshold of more than 5 mm [5]. On the other side, the sensitivity of pipelle examination varies greatly (from 62 to 99%), owing to the unevenness of the amount of collected specimens and the quality of the intrusion operation (due to pain, cervical stenosis and lack of operational experience).

Cervical mucus is secreted mainly by cervical endometrial cells and contains some fluid and tissue components from the uterus and fallopian tubes. Because of the anatomical continuity of the uterine cavity with the cervix, cervical mucus samples represent a unique opportunity to detect signs of disease shed from the upper genital tract. In Grande et al.’s study, inflammatory proteins were highly expressed in the cervical mucus of infertile women with ovarian endometriosis cysts [6]. In cancer screening, Rocconi et al. analyzed the cervical mucus proteome used for ovarian cancer screening [7]. Therefore, the lesions from the uterine cavity may be detected by some specific components in cervical mucus.

Engrailed-2(EN2) protein is a transcription-related factor that contributes to translational regulation [8]and can be detected in bodily fluids. Abnormal expression of EN2 has been observed in a variety of human cancers, such as bladder cancer, ovarian cancer, and breast cancer [9–12]. The EN2 protein content has been used as a urine detection indicator for early identification of prostate cancer through non-invasive testing, although its specificity is still controversial [13–16]. It is still unknown whether the expression of EN2 protein in cervical mucus can be used as a non-invasive detection for endometrial cancer.

We measured the EN2 content in cervical mucus of patients with different endometrial statuses and determined the diagnostic threshold, explaining the reasons for the different EN2 content in cervical mucus in different groups of patients through tissue and cell experiments.

## Methods

### Patients

From June 2019 to December 2020, a total of 133 patients visited Beijing Obstetrics and Gynecology Hospital and were included in this study. All tissue and cervical mucus samples were collected with informed consent according to the requirements of the Research Ethics Committee of Beijing Obstetrics and Gynecology Hospital, Capital Medical University (REC No: IEC-B-03-VO1-FJ1) and conducted between June 2019 and DEC 2020.

The inclusion criteria were as follows: (1) women aged 35–75 years; (2) patients willing to participate in the research. The cases were analyzed according to postoperative pathologic diagnosis. According to pathologic diagnosis, the test cases were divided into the following groups: EC group, EIN group(endometrial intraepithelial neoplasia), benign lesions group(myoma, benign ovarian cyst, endometrial polyps, etc.).We selected the routine physical examination women who met the following criteria into the normal control group. The inclusion criteria were as follows: (1) women aged 35–75 years; (2) women willing to participate in the study; (3) no abnormal bleeding symptom; (4) normal cervical Thinprep cell test (TCT) report; (5) normal endometrium thickness and no gynecologic tumor was found by TVS examination. Sexual activity, vaginal lavage, and drug application is prohibited within 24 h before sampling.

All postoperative pathological specimens of all patients were diagnosed by two or more senior pathologists. If the patient is diagnosed with endometrial cancer, surgical treatment is performed according to the NCCN guidelines, and the staging of endometrial cancer is determined based on the condition of all postoperative specimens. Based on the postoperative pathological results, we divided the patients into the endometrial stage Ia group, endometrial cancer ≥ IB stage group, EIN group, benign group, and control group.

### Ca125

The data came from the medical record system of Beijing Obstetrics and Gynecology Hospital. CA125 was recorded when the patient underwent a serological examination at our hospital. Before treatment, the patient extracted 3 ml of fasting venous blood without using a centrifuge and centrifuged at a speed of 3000r/min for 5 min for serum separation. The CA125 level was measured using electrochemiluminescence method, and all operations were strictly carried out according to the instructions of the reagent kit. The reagent kit was purchased from Beijing Jinhui Zhicheng Medical Technology Co., Ltd.

### Cells

Two human endometrial cancer cell lines were used: HEC1B and KLE, and the cervical adenocarcinoma cell line was used as a control cell line. These three cell lines were obtained from the China National Infrastructure of Cell Line Resource. The cell lines were authenticated by the supplier using DNA short-tandem repeat analysis. HEC-1B cells were derived from moderately differentiated endometrial adenocarcinoma, whereas the KLE cell line was derived from poorly differentiated EC and did not show ER or PR expression. All human cell lines were adherent and were maintained according to the supplied protocol. KLE cells were maintained in Dulbecco’s modified Eagle’s medium (DMEM)/F-12 (Sigma-Aldrich, Darmstadt, Germany), while HEB1C cells were maintained in modified Eagle medium (MEM) (Sigma-Aldrich, Darmstadt, Germany). The two cell lines were supplemented with 10% FBS (Gibco, Carlsbad, CA, USA) and 50 IU/mL penicillin–streptomycin. HeLa cells were cultured in DMEM supplemented with 10% FBS and antibiotics (100 U/mL penicillin G and 100 µg/mL streptomycin) at 37 °C in a humidified atmosphere of 5% CO2.

### Mucus sample collection

Mucus samples were obtained through vagina, by ophthalmological cotton swabs. The swabs were gently placed into the cervical canals and waited for 20 s to obtain cervical mucus, using swabs itself rather than aspiration to collect mucus from the canals to the greatest extent. Sampling was performed by two senior gynecologists, especially to avoid bleeding from mucosal contusion. After the operations above, the swab is placed in a cryogenic vial, numbered and immediately stored in liquid nitrogen.

### Mucus concentrations detected using ELISA

Mucus samples were transferred to the laboratory for testing by professional experimenters. The tests were conducted in a blind method, and the clinical data of the test samples were not known to the experimenters. Buffer solution (300 µL) was then added. After full absorption, the mixture was centrifuged at 1200 rpm for 15 min. The procedure was repeated twice. The supernatant of mucus was obtained by centrifugation. The levels of EN2 in the cervical mucus were quantified using ELISA, and according to the manufacturer’s instructions (Abcam, Cambridge, MA, USA). The tissue was ground and centrifuged. The supernatant of the tissues was measured as described above. All measurements were performed in triplicate to minimize the influence of technical error and intra-assay variation. All specimens were tested in triplicate, and the results were averaged for statistics.

### Tissue sample collection

We collected endometrial cancer lesions, endometrial tissue without endometrial cancer infiltration, and cervical tissue from 9 surgically treated endometrial cancer patients. Cancerous, para-carcinoma, and cervical tissues were obtained from patients with EC. We collected endometrial and cervical tissues from 15 patients who underwent hysterectomy for benign lesions such as uterine fibroids and ovarian cysts. Tissue samples were collected from inpatients who underwent hysterectomy within 15 min.All samples were stored in liquid nitrogen.

### Immunohistochemistry

Cells were plated in 12-well plates overnight, then fixed with 4% polyformaldehyde solution for 10 min and then with 0.1% Triton X-100 in phosphate-buffered saline (PBS) (Gibco, Carlsbad, CA, USA) for 10 min. Fixed cells were washed three times with PBS and incubated with 3% BSA in PBS for 1 h, and all steps above were performed at 20 ± 5 ℃.

Tissue samples were routinely fixed and embedded in paraffin wax. After antigen retrieval and blocking for 30 min, the slides were incubated with anti-human antibody against EN2 (Novus, Florida, USA) overnight at 4 °C.

EN2 staining was mainly nuclear. Levels of EN2 expression were scored based on staining intensity and distribution. The numbers of EN2 + cells were counted visually in five random microscopic fields at 200 × magnification for each biopsy by two blinded people. The EN2 scores were as follows: 3 + (positive/aberrant), complete and intense circumferential nuclear staining in > 50% of tumor cells; 2 + (equivocal), circumferential, incomplete and/or weak/moderate nuclear staining in > 25% tumor cells, or complete and circumferential intense nuclear staining in ≤ 25% tumor cells; 1 + (negative), no staining or incomplete and faint/barely perceptible nuclear staining in ≤ 10% tumor cells.

### Immunofluorescence

KLE, HEC1B, and HeLa cells were fixed in PFA for 20 min and transferred to 0.2% TritonX-100 overnight. They were then blocked with goat serum and incubated with 1:250 rabbit anti-EN2 antibody (Novus, Florida, USA). After washing with PBS, the preparations were incubated for 2 h with anti-rabbit IgG fluorescein isothiocyanate (FITC) conjugate (Novus, Florida, USA) 1:1000. Sections were washed and counterstained with 1 µg/mL of phalloidin, washed again, and then mounted in DAPI. All sections were visualized with an Optiphot Epifluorescence Microscope (Olympus, Middlebush, NJ, USA).

### RT-PCR

Real-time PCR was used to measure the mRNA levels of EN2 in cells. TRIzol reagent (Invitrogen, Carlsbad, California, USA) was used to extract total RNA from cells. The RNA samples were assessed for their purity and concentration using spectrophotometry. Reverse transcription (RT) was performed using the Reverse Transcription System (A3500, Promega, Madison, WI, USA) according to the manufacturer’s instructions. Real-time PCR was performed based on the detected fluorescence with SYBR Premix Ex Taq (Thermo Fisher Scientific Inc., Waltham, MA, USA). PCR was performed using specific primers synthesized by Tianyihuiyuan Biological Technology Co., Ltd. (Beijing, China). Relative mRNA expression levels were calculated using REL = 2-ΔΔCt, where ΔCt = Ct target gene – Ct GAPDH. All experiments were performed in triplicate.

### Statistical analysis

SignmaStat(version3.5;Sigma), SPSS software (version 23.0; SPSS Inc., Chicago, IL) and GraphPad Prism 8.0 ( La Jolla, CA, USA) were used to perform the statistical analyses. The means and standard deviations for the variables were calculated. Student’s *t*-test or one-way analysis of variance (one-way ANOVA) was used when the data met the assumption of normality; otherwise, non-parametric tests were used for the comparisons. Statistical significance was set at $\alpha$ < 0.05. GraphPad Prism software package was used to construct graphs and for statistical calculations. The significance level for all tests was set at 5%.

## Results

### EN2 protein levels were significantly increased in cervical mucus in the EC patients

133 samples (patient characteristics are shown in Table 1) were included in our study, Including 39 patients with stage IA endometrial cancer, 16 patients with stage IB and above, 16 patients with EIN (including 9 EINIs and 7 EINII-IIIs), 28 patients with benign endometrial lesions, and 34 patients with normal endometrium. Among them, the number of postmenopausal patients in the EIN group was significantly lower than that in the other groups, and there was no significant difference between the other groups. The proportion of CA125 elevation in patients with endometrial cancer staging higher than IB stage was significantly higher than that in other groups, and there was no significant difference between other groups, indicating that Ca125 is an effective biomarker for screening endometrial cancer above IB stage.

**Table 1:** Basic characteristics of the patients in the study

	Overall	EC stage Ia	EC ≥ stage Ib	EIN	Benign	Normal	*P*
N	133	39	16	16	28	34	
Age / Mean ± SD	52.0 ± 10.5	54.1 ± 10.5	55.5 ± 9.2	46.0 ± 12.7	51.5 ± 10.1	51.2 ± 9.7	0.069
Menopausal / *n* (%)	73 (54.9)	22(56.4)	10(62.5)	3 (18.8)	14 (50.0)	24 (70.6)	0.014
CA125 Elevated / *n* (%)	12(9.7)	1(8.3)	7(43.8)	0 (0.0)	4 (14.3)	0(0.0)	0.020
EN2	345.5 ± 234.7	563.3 ± 119.8	599.8 ± 132.2	305.0 ± 198.6	153.5 ± 106.2	153.1 ± 107.5	< 0.001

Statistical significance in EN2 concentration is shown between EC vs. EIN (*P* < 0.001, Table 1), EIN vs. benign and control (*P* < 0.001), and EC vs. benign and control(*P* < 0.001), while no statistical significance is indicated between EC Ia vs. EC Ib and above(*P* = 0.342), and Benign vs./ Control group(*P* = 0.988).

Due to significant differences in menopausal status among different groups, we compared the EN2 values in cervical secretions of patients with different menopausal statuses in the same group to exclude the influence of menopausal status on EN2 values. Since only three EIN patients experienced menopause, we did not compare the impact of menopause on the cervical EN2 values of EIN patients. There was no difference between the groups in age at diagnosis, years of postmenopausal. There was no significant difference in EN2 level between the non-menopausal and menopausal samples within the group (Table 2), suggest that menopausal status has no significant impact on EN2.

**Table 2:** EN2 protein concentration in cervical mucus between the menopausal and non-menopausal samples within the group

Group	N	Mean EN2(ng/ml)	Mean EN2 (ng/ml)		*P* value
			Menopausal	Non-menopausal	
EC	55	573.9 ± 123.4	559.4 ± 134.8	593.2 ± 109.2	0.33
EIN	16	305.0 ± 198.6	349.8 ± 229.8	294.71 ± 189.2	–
Benign	28	153.5 ± 106.2	127.0 ± 96.2	180.0 ± 112.5	0.51
Control	34	153.0 ± 107.5	149.9 ± 109.7	160.7 ± 107.4	0.88

**Fig. 1:**
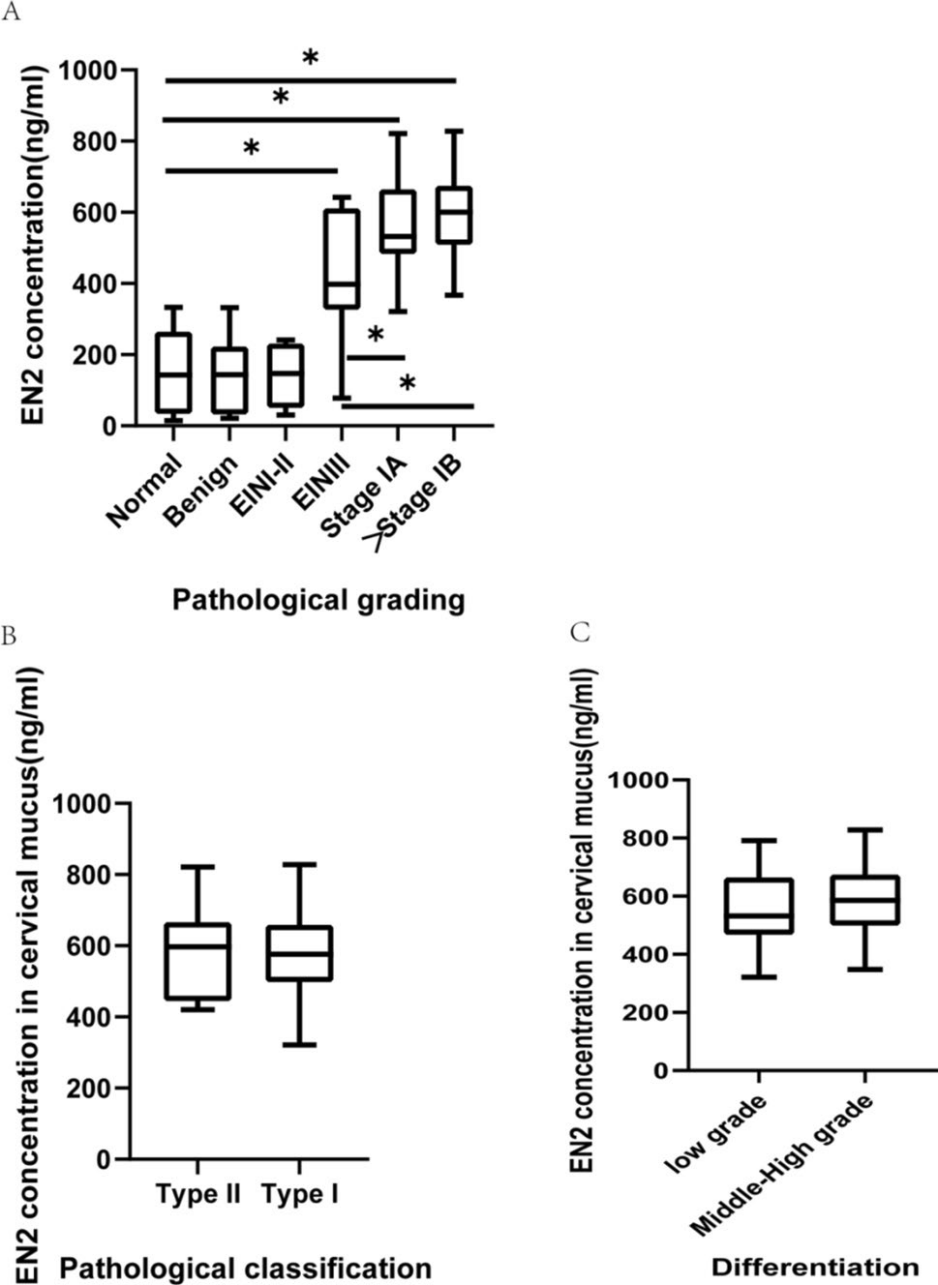
The comparison of mean EN2 concentrations in cervical mucus. (A:Normal:Benign *P* = 0.477;Normal:EINI-II *P* = 0.885;Normal:EINIII:*P* < 0.001;Normal:Stage 1a *P* < 0.001;Normal:Stage 1b *P* < 0.001;Benign:EINI-II *P* = 0.880;Benign:EINIII *P* < 0.001;Benign:Stage 1a:*P* < 0.001;Benign:Stage 1b:*P* < 0.001;EINI-II:EINIII *P* < 0.001;EINI-II:Stage 1a *P* = 0.002;EINI-II:Stage 1b *P* < 0.001; Stage 1a:1B P = 0.305)

We measured the expression level of EN2 in the cervical mucus of patients in different groups. The mean cervical mucus EN2 concentration(573.9 ± 123.4 ng/mL) in the EC group significantly higher than benign lesion group(153.5 ± 106.2 ng/mL) and control group(53.0 ± 107.5 ng/mL) (Fig. 1A, *P* < 0.001). In addition, the EN2 protein concentration of EIN3 case (428.8 ± 185.6 ng/ml) was significantly higher than that of EIN I-II case samples(145.9 ± 82.2 ng/ml, P < 0.001), while the EN2 protein concentration of EIN I-II case was similar as the normal control group (153.050 ± 107.5 ng/ml, *P* = 0.885) and the Benign group (153.5 ± 106.2 ng/ml, *P* = 0.880)(Fig. 1A). The EN2 level in the cervical mucus of FIGO stage 1a EC cases were significantly higher than that of EIN3 cases. There was no significant difference in the expression of EN2 protein in cervical mucus between Stage IA patients (563.3 ± 119.8 ng/ml) and above Stage IB patients(599.8 ± 13.2 ng/m, *P* = 0.305). We grouped patients based on pathological type and differentiation degree, and compared the differences in EN2 expression in cervical mucus between groups(Fig. 1B classification; Fig. 1C differentiation). There was no evidence that pathological classification(Type I:572.0 ± 121.8 ng/ml; Type II:585.3 ± 140.7 ng/ml, *P* = 0.781, Fig. 1B) and differentiation of EC(Low grade:551.0 ± 126.9 ng/ml, Middle-High grade:585.1 ± 121.9 ng/ml, *P* = 0.341, Fig. 1C) has an influence on the dose of EN2.

### Cervical mucus EN2 concentrations are predictive for EIN and EC

**Fig. 2:**
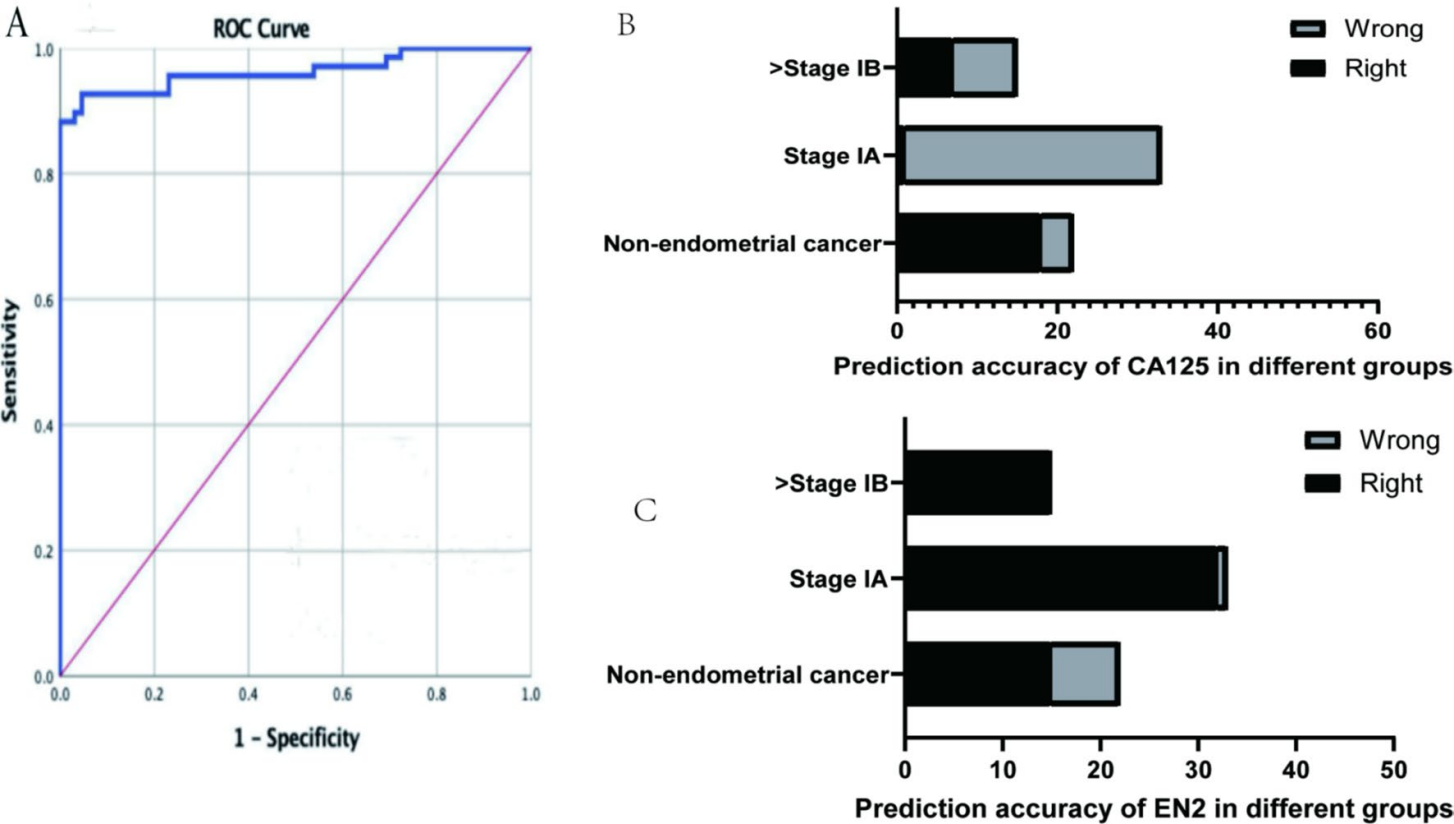
Receiver operating characteristic (ROC) curves of the prediction performance of EN2 protein

The difference in cervical mucus EN2 concentrations in patients with and without EC indicated potential diagnostic value. We use the concentration of EN2 in cervical mucus to predict whether patients have endometrial lesions above EINIII. A ROC analysis of these data has an AUC of 0.963 (Fig. 2A), indicating that the concentration of EN2 is a good predictive value The ROC analysis indicated that the optimal cervical mucus EN2 concentration threshold for EC versus non-cancer was 321.1 ng/ml to maximize the sensitivity and acceptable optimized specificity of the test. Using this cut-off gives a sensitivity for total EINIII and EC of 92.6% with a specificity of 95.4%. The sensitivity for EINIII and all EC is 88.9% and 100% respectively.

We collected serum CA125 values from 15 patients in the Stage IB group and 33 patients in the Stage IA group. When CA125 was higher than 35U/ml, it was considered to indicate endometrial lesions. The sensitivity of EN2 protein in each stage is significantly higher than that of Ca125 in the same stage(> Stage IB 100%:46.7%; Stage IA 97.0%:3.0%,*P* = 0.000, Fig. 2 BC).

### EN2 protein over-expression in human EC tissue

**Fig. 3:**
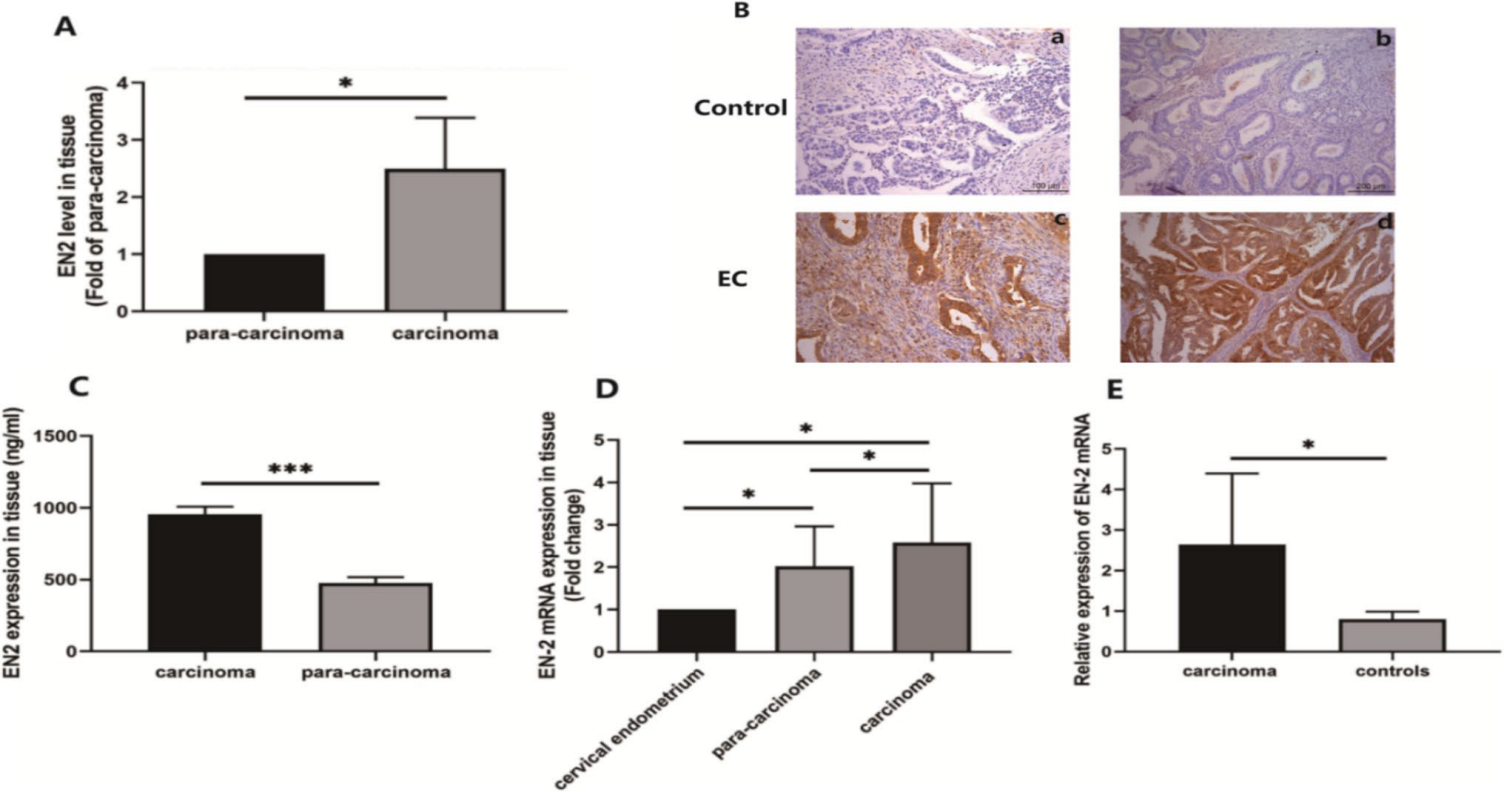
RT-PCR measured EN2 protein in para-carcinoma and carcinoma (Fig. 3A: *n* = 9, *P* = 0.019, P: para-carcinoma, C: carcinoma). EN2 staining of EC and benign control sections with an anti-EN2 antibody and HE staining, EC tissues showed strong positive (3 +) EN2 protein expression, but the benign control tissues were negative (Fig. 3B, *n* = 15, EN2 staining shows brown, EC tissue positive cells > 50% 3 + , and the benign control tissue did not show brown). ELISA indicated that the difference in EN2 protein between the carcinoma and para-carcinoma tissues was significant (Fig. 3C, *n* = 15, *P* < 0.001). The level of EN2 expression detected using RT-PCR in carcinoma was higher than that in para-carcinoma, and the level in para-carcinoma was higher than that in the cervical endometrium (Fig. 3D, *n* = 15, *P* < 0.05). EN2 protein detected using RT-PCR in carcinoma cells was higher than that in controls (Fig. 3E, *n* = 15, *P* = 0.0357)

To investigate the reasons for the increased expression of EN2 protein in cervical mucus, we selected endometrial cancer lesions from the same patient, endometrial tissue without endometrial cancer infiltration, and cervical tissue for the study. RT-PCR indicated that EN2 expression in the carcinoma was much higher than in para-carcinoma tissues (Fig. 3A, *P* = 0.019). EC tissues showed strong positive EN2 protein expression, but the para-cancinoma tissues were negative (Fig. 3B). EN2 protein concentration in tumor tissue detected using ELISA was significantly higher than that in para-carcinoma tissues (Fig. 3C, *P* < 0.001). The level of EN2 detected using RT-PCR in carcinoma was higher than that in para-carcinoma, and the level in para-carcinoma was higher than that in the cervical endometrium (Fig. 3D, *P* < 0.05). EN2 protein detected using RT-PCR in carcinoma was higher than that in benign controls (Fig. 3E, *P* = 0.0357). The results suggest that the increase in EN2 protein content in cervical mucus may be caused by the high expression of endometrial cancer cells.

### EN2 protein overexpression in EC cell lines

**Fig. 4:**
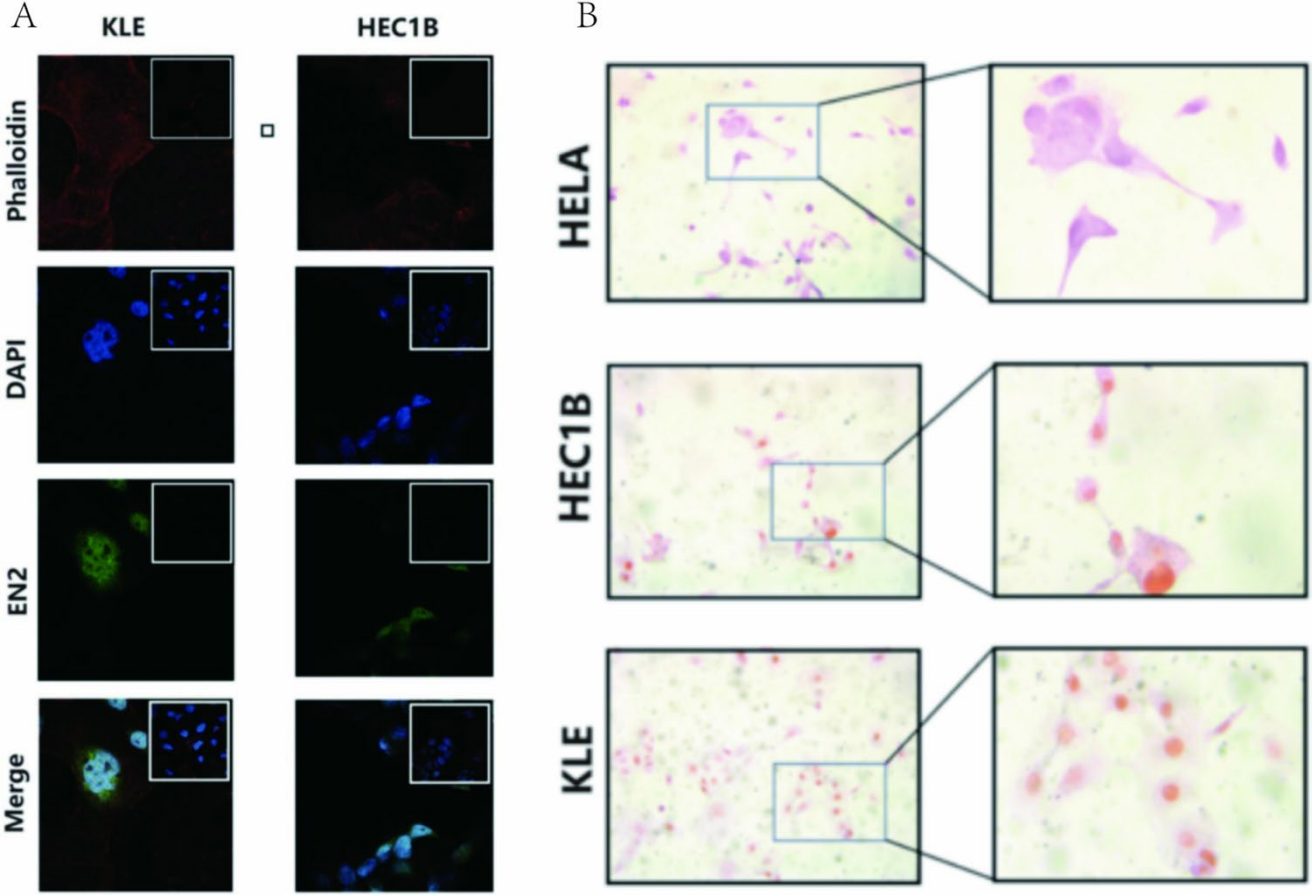
EN2 protein was expressed in endometrial cancer cell lines

To evaluate the expression level of EN2 in endometrial cells, we selected the HEC-1-B (hormone related) type I endometrial cancer cell line and KLE type II endometrial cancer cell line for our study. Confocal microscopy showed that EN2 appeared to be predominantly expressed within the nucleus of the two cell lines (Fig. 4A). Enzymatic immunohistochemistry examples of EC stained for EN2 in cell lines demonstrated that EN2 was expressed in the nucleus (Fig. 4B).

## Discussion

With the development of molecular biology technology, molecules (e.g., mutated DNA fragments, miRNA, or proteins) differently expressed in and released from tumor cells can be detected using various molecular biochemical techniques in different types of body fluids, which is known as liquid biopsy. Liquid biopsy can be applied in cancer screening or diagnosis to avoid or minimize invasive operations. These studies are focused on tumors that exist in the external connecting parts. Lung cancer patients can be diagnosed by mi RNA in sputum or serum [17]. pancreatic cancer research based on saliva and ascites is also underway [18].

In this study, we found that the concentration of EN2 protein in the cervical mucus of EINIII and EC cases was significantly higher than that in EINI-II cases, benign cases and normal control cases. The ROC analysis indicated that the optimal cervical mucus EN2 concentration threshold for EINIII and EC was 321.1 ng/mL, with a sensitivity of 92.6% for total EINIII and EC and a specificity of 95.4%. The sensitivity of the EINIII and stage 1A EC cases was 88.9% and 100% respectively. The significantly increased level of EN2 protein in the early cases (EINIII and stage IA) show a good test efficacy. Both sensitivity and specificity were remarkably superior to existing EC tumor markers, such as CA125 and HE4. Furthermore, there was no difference in EN2 test results between menopausal and non-menopausal cases, so it can also be used in postmenopausal patients. For cases with different pathological types, the level of EN2 was totally increased. This means all EC cases including type I and type II can be screened out. Therefore, EN2 protein is suitable for screening biomarker.

High concentrations of EN2 protein in the cervical mucus of EC patients are derived from two possible pathways. One possible explanation for this phenomena is that the EN2 protein in cervical mucus is from exfoliated EC cells. Exfoliated and dissolved tumor cells can release EN2 protein. EN2 protein can be excreted through the cervical canal, where it can be mixed in the cervical mucus. If EC cells express a high level of EN2 protein, high level of EN2 protein in the cervical mucus of EC patients can be detected. We found that the EN2 protein was overexpressed in the KLE and HEB1C cell lines compared with that in the HeLa cell line as a control. However, no significant difference was found between the KLE and HEB1C lines, which represent moderately and poorly differentiated EC, respectively. Meanwhile, our study observed that the positive reaction of en2 protein in immunohistochemical staining section of endometrial cancer tissue was significantly stronger than that of normal endometrial tissue. EN2 protein was highly expressed in EC tissues compared with that in the paracancerous and endocervical tissues taken from EC patients. These findings confirm that the EN2 protein is highly expressed in endometrial cancer cells, regardless of the degree of cell differentiation. Several studies about human epithelial malignant tumors have also demonstrated high expression of the EN2 gene, such as prostate cancer, bladder cancer, ovarian cancer, and breast cancer [8–12]. Recent studies have shown that the EN2 gene may play an important role in the proliferation and migration of tumor cells [8, 10, 11].

Another possible theory is that EC cells themselves can secrete this EN2 protein and mixed into cervical mucus. At present, EN2 mRNA is considered a transcription factor. A recent study showed that EN2 protein can be secreted from cancer cells and taken up by other cells, providing a typical example of this phenomenon. Richard Morgan reported that EN2 is expressed in, and secreted by primary or recurrent bladder cancer. EN2 expression and secretion may be an early event in bladder tumorigenesis independent of the specific molecular dysregulation associated with bladder cancer [14]. Another study using immunofluorescence antibody reported that prostate cancer cells secrete fluorescence-labeled EN2 protein through microbubbles. Prostate cancer cells and normal prostate cells can absorb these EN2 proteins in running microcapsules. When cancer cells in their dormant phase absorb these EN2 proteins, they are reactivated, changed in shape, or fused together [19]. EN2 protein expressed and secreted through prostate cancer cells can be released into prostate cancer acini and catheters through prostate secretions; therefore, high concentrations of EN2 protein can be detected in the urine of prostate cancer patients. According to the discovery of EN2 research, we have reason to believe that abnormally high concentrations of EN2 protein in cervical mucus are closely related to EC cells, and EC can be identified by detecting abnormally high EN2 protein concentrations.

An ideal early diagnostic biomarker would be a serological or secretion-based biomarker with noninvasive collection, and the cost would be parity. EN2 protein may have potential as a novel biomarker for EC. In the future, we envisage EN2 protein is more suitable for rapid qualitative screening test. Asymptomatic women among high-risk population such as female with sex hormone therapy history or family history of cancer (e.g., Lynch syndrome) need screening. Since the sensitivity of EN2 detection for early endometrial lesions changes (EINIII) is 89.2%, EC screening in adult women is applicable. Meanwhile, because the specificity of EN2 detection for EC is 95.4%, EN2 negative can reduce unnecessary invasive operations such as hysteroscopy and diagnostic curettage for patients with abnormal endometrial thickness detected via TVS. The limitation of our research is the preliminary small sample study. In the future, we plan to carry out a large sample prospective clinical study to further confirm the threshold of EN2 protein concentration in cervical mucus and evaluate its sensitivity and specificity as an EC tumor biomarker.

Endometrial cancer mainly occurs in perimenopausal and postmenopausal women [20]. Although there was no significant age difference between the groups in our study (*P* = 0.069), the average age of the endometrial cancer group was significantly higher than that of the EIN group. As this study was a small-scale experiment, there was no strict age matching for patients. It is currently unclear whether age has an impact on the expression level of EN2 protein in cervical mucus. Another age-related factor is whether the patient has already reached menopause. In our study, menopause had no significant effect on the expression of EN2 protein in the cervical mucus of patients, indicating that this method can be applied to all perimenopausal women without considering the effect of hormone levels on EN2 protein expression. CA125 is a commonly used tumor marker for detecting the occurrence, metastasis, and prognosis of cancer [21, 22]. Elevated CA125 is not very common in early tumors, and there are currently many studies monitoring CA125 in combination with other tumor markers [23, 24]. In this study, only one patient in stage Ia had elevated CA125 expression, while the proportion of patients in stage Ib with elevated CA125 reached 43.8%. However, if the EN2 content in cervical mucus was used to classify patients, the early accuracy was significantly higher than CA125. We did not further combine CA125 results with EN2 protein expression.

This study has certain limitations. We did not conduct a more in-depth analysis of the mechanism of EN2 in the occurrence and development of endometrial cancer, nor did we characterize the changes in EN2 expression on the characteristics of endometrial cancer cells or tissues. In this study, the average age of the patients we included was over 50 years old. As the effect of age on EN2 protein is not yet clear, it is unknown whether EN2 protein measurement has the same efficacy for younger endometrial cancer patients. In addition, we included specimens from 133 patients, which is still a small-scale study and requires larger-scale research to determine the value and cutoff value of cervical mucus EN2 protein.

In conclusion, overexpression of EN2 in EC tissue may play an important role in tumor invasion and progression. Because of the advantages of EN2 protein in EC detection (e.g. remarkably high concentration, easy collection, non-invasive, cheap, and reliable experimental techniques), it is reasonable to believe that these findings may provide a new method for the early detection of EC.

## Data Availability

The datasets analyzed during the current study are available from the corresponding author upon reasonable request.
